# Retraction: LBX2‐AS1 promotes ovarian cancer progression by facilitating E2F2 gene expression via Mir‐455‐5p and Mir‐491‐5p sponging

**DOI:** 10.1111/jcmm.18563

**Published:** 2024-08-01

## Abstract

J. Cao, H. Wang, G. Liu, R. Tang, Y. Ding, P. Xu, H. Wang, J. Miao, X. Gu, and S. Han, ‘LBX2‐AS1 promotes ovarian cancer progression by facilitating E2F2 gene expression via Mir‐455‐5p and Mir‐491‐5p sponging’, *Journal of Cellular and Molecular Medicine* 25, no. 2 (2021): 1178–1189, https://doi.org/10.1111/jcmm.16185.

The above article, published online on 20 December 2020 in Wiley Online Library (wileyonlinelibrary.com), has been retracted by agreement between the journal Editor‐in‐Chief, Stefan Constantinescu; The Foundation for Cellular and Molecular Medicine; and John Wiley and Sons Ltd. The retraction has been agreed following an investigation into concerns raised by a third party, which revealed inappropriate duplications of image panels (Figure 2C, D, E and 5C) between this and other articles that were either previously published or published later in the same year. Thus, the editors consider the conclusions of this manuscript substantially compromised. The authors were informed of the decision to retract but did not respond to the retraction decision or the wording.

J. Cao, H. Wang, G. Liu, R. Tang, Y. Ding, P. Xu, H. Wang, J. Miao, X. Gu, and S. Han, ‘LBX2‐AS1 promotes ovarian cancer progression by facilitating E2F2 gene expression via Mir‐455‐5p and Mir‐491‐5p sponging’, *Journal of Cellular and Molecular Medicine* 25, no. 2 (2021): 1178–1189, https://doi.org/10.1111/jcmm.16185.

The above article, published online on 20 December 2020 in Wiley Online Library (wileyonlinelibrary.com), has been retracted by agreement between the journal Editor‐in‐Chief, Stefan Constantinescu; The Foundation for Cellular and Molecular Medicine; and John Wiley and Sons Ltd. The retraction has been agreed following an investigation into concerns raised by a third party, which revealed inappropriate duplications of image panels (Figure 2C, D, E and 5C) between this and other articles that were either previously published or published later in the same year. Thus, the editors consider the conclusions of this manuscript substantially compromised. The authors were informed of the decision to retract but did not respond to the retraction decision or the wording.

